# PrEP Use, Sexual Behaviour, and PrEP Adherence Among Men who have Sex with Men Living in Wales Prior to and During the COVID-19 Pandemic

**DOI:** 10.1007/s10461-022-03618-4

**Published:** 2022-02-19

**Authors:** D. Gillespie, Z. Couzens, M. de Bruin, D. A. Hughes, A. Jones, R. Ma, A. Williams, F. Wood, K. Blee, H. S. Bradshaw, R. Drayton, C. Knapper, K. Hood

**Affiliations:** 1 Centre for Trials Research, College of Biomedical & Life Sciences, School of Medicine, Cardiff University, Cardiff, Wales UK; 2 Public Health Wales NHS Trust, Cardiff, Wales UK; 3 Radboud University Medical Center, Institute of Health Sciences, IQ Healthcare, Nijmegen, Netherlands; 4 Centre for Health Economics and Medicines Evaluation, Bangor University, Bangor, Wales UK; 5 Policy, Research and International Development, Public Health Wales, Cardiff, Wales UK; 6 Imperial College London, London, England UK; 7 Division of Population Medicine and PRIME Centre Wales, College of Biomedical & Life Sciences, School of Medicine, Cardiff University, Cardiff, Wales UK; 8 Sexual Health Department, Glan Clwyd Hospital, Betsi Cadwaladr University Health Board, Rhyl, Wales UK; 9 Sexual Health Department, Singleton Hospital, Swansea Bay University Health Board, Swansea, Wales UK; 10 Sexual Health Department, Cardiff Royal Infirmary, Cardiff and Vale University Health Board, Cardiff, Wales UK; 11 Cordell Centre, Royal Gwent Hospital, Aneurin Bevan University Health Board, Newport, Wales UK

**Keywords:** HIV prevention, PrEP, Sexual behaviour, Adherence, COVID-19

## Abstract

We examined PrEP use, condomless anal sex (CAS), and PrEP adherence among men who have sex with men (MSM) attending sexual health clinics in Wales, UK. In addition, we explored the association between the introduction of measures to control transmission of SARS-CoV-2 on these outcomes. We conducted an ecological momentary assessment study of individuals in receipt of PrEP in Wales. Participants used an electronic medication cap to record PrEP use and completed weekly sexual behaviour surveys. We defined adherence to daily PrEP as the percentage of CAS episodes covered by daily PrEP (preceded by ≥ 3 days of PrEP and followed by ≥ 2 days). Sixty participants were recruited between September 2019 and January 2020. PrEP use data prior to the introduction of control measures were available over 5785 person-days (88%) and following their introduction 7537 person-days (80%). Data on CAS episodes were available for 5559 (85%) and 7354 (78%) person-days prior to and following control measures respectively. Prior to the introduction of control measures, PrEP was taken on 3791/5785 (66%) days, there were CAS episodes on 506/5559 (9%) days, and 207/406 (51%) of CAS episodes were covered by an adequate amount of daily PrEP. The introduction of pandemic-related control measures was associated with a reduction in PrEP use (OR 0.44, 95%CI 0.20–0.95), CAS (OR 0.35, 95%CI 0.17–0.69), and PrEP adherence (RR = 0.55, 95%CI 0.34–0.89) and this may have implications for the health and wellbeing of PrEP users and, in addition to disruption across sexual health services, may contribute to wider threats across the HIV prevention cascade.

**Supplementary Information:**

The online version contains supplementary material available at 10.1007/s10461-022-03618-4.

## Introduction

HIV pre-exposure prophylaxis (PrEP) involves the use of antiretroviral (ARV) medication for HIV-negative individuals to prevent HIV acquisition through high-risk sexual encounters [1]. Its use has been demonstrated to be highly effective in preventing HIV acquisition in several key populations in the context of large clinical trials [2–4] Furthermore, additional analysis of drug concentrations and medication use indicates that HIV-1 risk reduction was 96% and 99% for individuals taking PrEP four and seven days a week respectively, demonstrating its high levels of efficacy [5]. PrEP is now routinely available through healthcare providers in multiple countries worldwide, including Wales (UK), where oral PrEP (tenofovir-emtricitabine (TDF-FTC)) is licensed for daily use in people considered at risk of HIV-acquisition [6]. For PrEP to work as intended, it needs to be taken during periods where an individual is at-risk of acquiring HIV through condomless sexual contact [7].

Adherence to pharmaceutical regimen refers to studying ‘the process by which patients take their medication as prescribed’, and is comprised of treatment initiation (when the patient takes their first dose), implementation (the extent to which a patient's actual dosing corresponds to the prescribed dosing regimen), and persistence (the length of time between initiation and the last dose) [8]. Studying ‘PrEP implementation is particularly complex as it requires careful assessment of both PrEP intake as well as people’s sexual activity.

When TDF-FTC is prescribed for daily use, PrEP adherence can be calculated as ‘the % of days with correct dosing’, which only requires the measurement of medication use. However, other regimens can be followed and have a growing evidence-base (e.g. event-based dosing, which involves taking two pills as a single dose 2–24 h prior to condomless sexual intercourse, followed by one pill a day thereafter until two sex-free days have passed and is thus intrinsically aligned to risk exposure), particularly in men who have sex with men (MSM) [9]. Furthermore, sexual risk behaviour patterns can differ between and within individuals over time and arguably should be aligned to and considered in the context of risk exposure, regardless of regimen [10]. Hence, in relation to the expected prophylactic effects of TDF-FTC, it is most appropriate to define PrEP adherence as ‘the % of condomless sexual encounters adequately covered by PrEP’.

To date, cohort studies enrolling PrEP users and measuring adherence longitudinally in high-income countries have used a range of methods to measure PrEP adherence, including self-report [11] and dried blood spots [12]. A minority of studies measured adherence via a combination of medication use and risk exposure, with those that did doing so via self-report and tending to report lower levels of adherence than studies relying on medication use alone [13]. Electronic monitoring offers a means towards measuring day-to-day medication use (rather than overall consumption over a defined period) and hence may be both best placed to accurately measure implementation of PrEP use over time and align PrEP use with risk episodes, thus offering longitudinal insight into so-called “prevention-effective adherence” and enable understanding of the potential risk associated with non-adherence to PrEP [7]. Within the UK, there are no cohort studies that have measured PrEP use electronically, nor any that have measured adherence via a combination of medication use and risk exposure.

In the absence of such studies in a UK context, the current study set out to assess PrEP adherence in real-life using intensively sampled longitudinal data measuring both electronically-measured PrEP use and condomless sexual behaviour. We aimed to estimate levels of PrEP use, condomless sexual behaviour, and PrEP adherence (using a definition of adherence that aligned electronically monitored PrEP use data with condomless sexual behaviour) in real-life among people receiving TDF-FTC as HIV PrEP through sexual health clinics in Wales, UK.

During our study, the COVID-19 pandemic occurred and control measures were introduced to limit the spread of SARS-CoV-2, the respiratory virus that causes COVID-19. These measures included social distancing and advice to restrict journeys outdoors and physical contact with non-household members. Guidance was issued by the British Association for Sexual Health and HIV (BASHH) indicating that people should only have sexual contact with someone if they lived within the same household [14]. Furthermore, sexual health clinics limited contact with clients; in some cases suspending their PrEP service temporarily. The impact of pandemic-related control measures on PrEP use, sexual behaviour, and PrEP adherence among PrEP users living in Wales is as yet unknown, with initial analysis suggesting a marked reduction in condomless anal sex (CAS) in the short-term [15]. The impact of pandemic-related control measures on PrEP use and PrEP adherence is important to understand as it may highlight additional support needs PrEP users require during periods of major change or disruption to their lives. This paper will therefore also consider behaviours within this broader context and determine whether the introduction of control measures was associated with changes in PrEP use, CAS, and PrEP adherence.

## Methods

### Study Design and Participants

This ecological momentary assessment (EMA) [16] study was conducted in sexual health clinics across four health boards (geographically-defined administrative units responsible for planning and delivering health services in their area) offering PrEP in Wales, UK (a nation with a relatively low HIV prevalence of 0.08% in 2019 compared to a global HIV prevalence of 0.7% in the same year [17]). The clinics and health boards were selected for their geographical diversity, serving urban and rural populations. Participants were those in receipt of a prescription of TDF-FTC to prevent HIV-1 and aged at least 16 years. Both new and existing PrEP users were eligible for inclusion and participants were not excluded from the study if they adopted a non-daily regimen. Individuals were excluded if they lacked capacity to consent, were unable to provide a mobile telephone number linked to a smartphone, unable to use the Medication Event Monitoring System (MEMS) cap (described below), or unable to provide an e-mail address. The study was reviewed and approved by the Wales Research Ethics Committee 3 (Reference Number: 19/WA/0175).

The study is reported in accordance with STROBE [18], EMERGE [19], and CREMAS [20] guidelines for the reporting of observational, medication adherence, and EMA studies. Furthermore, we have used the TEOS framework for our operational definition of PrEP use [21].

### Procedures

Participants attending sexual health services to obtain a PrEP prescription were consecutively approached about the study by their treating clinician. Participants who expressed interest were screened as eligible, consented to the study, and completed a questionnaire on a tablet. The questions covered sociodemographic details, health beliefs and behaviours, sex and relationships, the presence and interference of symptoms commonly attributed to PrEP use (identified from the summary of product characteristics for TDF-FTC), and healthcare contacts. Participants were then supplied with a MEMS cap (a medication bottle cap containing an electronic monitor which records the date and time of each opening) [22, 23], shown how to use it (i.e. they were instructed to only open and replace the cap when they were taking their PrEP medication). One-week following recruitment, and thereafter weekly until study participation ended, participants were e-mailed a link to an online survey about condomless anal and vaginal sex (CAS and CVS respectively) during the preceding week. The survey was designed to be completed via mobile, tablet, or computer web browsers (See Table SI in online supplementary material). Reminder e-mails were sent to participants if they had not completed the survey within two days. Follow-up questionnaires, including the same questions asked at recruitment in addition to questions on self-reported PrEP use, were administered at three time points aligning to PrEP clinic follow-ups (approximately three months apart—herein Follow-ups 1, 2, and 3). These initially took place in person within a sexual health clinic. However, following the introduction of measures to control the spread of SARS-CoV-2, these were conducted over the telephone or online. At follow-up time points, participants were asked whether they were experiencing any difficulties using their MEMS caps and reminded how to use them. Data from PrEP clinic notes were also extracted covering the study period.

### Outcomes

The primary outcomes were:

Daily PrEP use over time, as measured via MEMS-caps [7, 8, 24]. We calculated the percentage of observed days on which PrEP was taken as the total number of days on which PrEP was used (i.e. the MEMS cap was opened at least once) divided by the total number of days over participants were observed during the study. This outcome was measured and reported for all participants (regardless of PrEP regimen followed).

Daily CAS over time was measured via brief, weekly online questionnaires. These captured daily data on whether CAS occurred, the number of times it occurred, and the number of partners with whom it occurred. Questions were informed by previous research (specifically, recommendations for using short recall periods [7, 25] and considering not only if a CAS episode occurred but also the number of times it occurred and number of partners with whom it occurred [26]) and developed by the research team and a stakeholder group comprising current PrEP users, PrEP providers, and individuals involved in sexual health advocacy and policy. They were also piloted prior to use (see Table SI for the list of questions). We calculated the percentage of observed days on which CAS occurred as the total number of days on which at least one CAS episode occurred divided by the total number of days over participants were observed during the study, as well as the mean number of episodes and mean number of partners on a given day. Similar to PrEP use, this outcome was measured and reported for all participants.

As a secondary outcome, we used a working definition of “adherence to daily PrEP”, which considered coverage of CAS episodes by sufficient doses of daily PrEP and was calculated as the percentage of all days on which CAS occurred. For a CAS episode, we considered a sufficient dose of PrEP to comprise at least 3 days of PrEP use prior to the CAS episode and at least two days of PrEP use following a CAS episode [27]. We excluded participants from this analysis if it was indicated in their clinic notes that they were following an event-based regimen.

### Sample Descriptives

We collected sociodemographic characteristics of participants, in addition to HIV risk perceptions, stigma, health beliefs, STI diagnoses and treatments, and healthcare resource use. These are reported as frequencies with percentages and medians with interquartile ranges.

### Statistical Analysis

The original sample size calculation was based on recruiting 60 participants, with each participant followed up for at least seven months. Assuming some drop out and discontinuation of PrEP use (i.e. an average of 160 days of PrEP use data per individual), this would provide approximately 84% power with a two-sided alpha of 0.05 to estimate an overall average probability of PrEP use on a given day of 0.7, [9] assuming an intra-cluster correlation coefficient of 0.6 [28]. However, we ended up following up participants for approximately nine-months (incorporating up to four clinic visits per participant in total) and thus increasing the potential number of observations available per participant.

Our original plan involved modelling our outcomes across all time points. However, the control measures introduced to reduce transmission of SARS-CoV-2 meant that separate consideration of time periods defined by the introduction of control measures would be more meaningful. Observations prior to the introduction of control measures are likely to be more reflective of natural behaviour (and hence reflective of the original aims when first conceptualising the study) and observations following their introduction allow for an investigation of the impact of the pandemic on our outcomes.

To model PrEP use over time, we fitted a two-level mixed-effects logistic regression model (PrEP use—yes/no), which accounted for repeated observations within individuals. We allowed for non-linear patterns in PrEP use over time by modelling time as a restricted cubic spline with four knots [29].

To model sexual behaviour over time, we fitted the following models: (i) a two-level mixed-effects logistic regression model to CAS (yes/no); (ii) a two-level mixed-effects ordered logistic regression model to the number of times a participant engaged in CAS (0/1/2/3 or more); (iii) a two-level mixed-effects ordered logistic regression model to the number of CAS partners (0/1/2 or more). In all three models, time was modelled as a restricted cubic spline with five knots [29]. Time was defined as time since UK control measures were introduced in response to the COVID-19 pandemic (hereafter “control measures”, where time zero was 16/03/2020). This simultaneously facilitated investigation of PrEP use and sexual behaviour over chronological time and supported our exploration and interpretation of the impact of the introduction of pandemic-related control measures across Wales. All models included a variable indicating whether an observation occurred during the intra-pandemic period as a random effect.

See page 2 of the supplementary material for further models fitted to investigate PrEP use and CAS over time.

To examine PrEP adherence over high- risk sexual encounters, we fitted log-binomial regression models to the number of CAS episodes covered by PrEP, with the number of CAS episodes as the exposure and whether the observations occurred during the pre- or intra-pandemic period included as a covariate. We calculated cluster robust standard errors to account for repeated observations within individuals. We conducted a sensitivity analysis whereby we excluded participant time periods (e.g. PrEP adherence data covering study entry to follow-up 1, follow-up 1 to follow-up 2, etc.) where the MEMS cap may have been used inconsistently (see page 2 of the supplementary material for further details).

Statistical analyses were conducted using Stata (v16.1) [30].

## Results

### Participant Recruitment

**Fig. 1:**
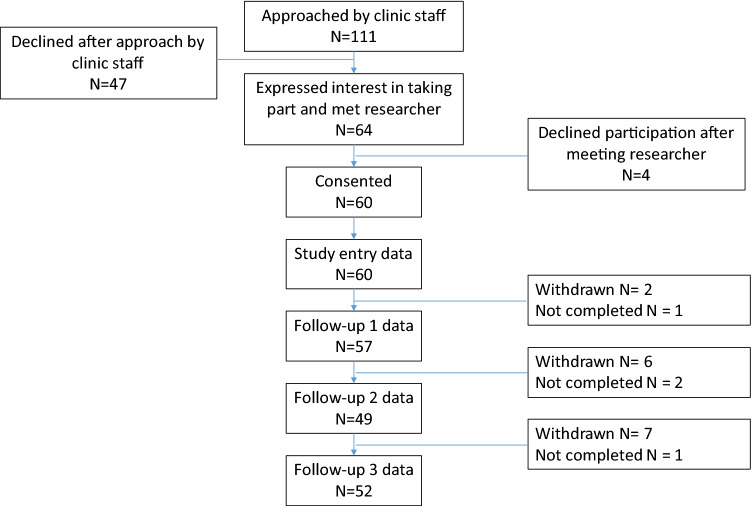
Participant flow diagram

Between 24/09/2019 and 27/01/2020, clinic staff approached 111 individuals about the study across 23 clinic sessions, of whom 64 agreed to speak to a researcher and 60 were recruited (Fig. 1).

### Participant Characteristics

All recruited participants were cis gender and male. The majority identified as white British (53/60, 88.3%), the median age was 35.5 years (IQR 28 to 46 years), 42/60 were in full-time employment (70.0%), and 29/60 were educated to degree level or equivalent (48.3%). New PrEP users (i.e. those who were recruited on the same day they were first prescribed PrEP) accounted for 6/60 of the sample (10.0%). The majority of participants identified as a gay man (56/60, 93.3%), and all but one participant had sex exclusively with other men. Most participants were single at the time they were recruited (46/60, 76.7%). Overall, 27/60 had a documented chronic health condition in their sexual health clinic notes (45.0%) (Table 1). Table SII describes health beliefs and behaviours at study entry. Table SIII describes STI diagnoses and healthcare resource use in the 3 months prior to study entry.

**Table 1:** Participant characteristics at study entry

Variable	Overall [N = 60]	
	n	%
*Sex*		
Male	60	100.0
*Gender*		
Cis gender	60	100.0
*Ethnicity*		
White British	53	88.3
White European	4	6.7
White	1	1.7
African	1	1.7
White and Black African	1	1.7
*Employment status*		
Full-time employed	42	70.0
Part-time employed	6	10.0
Casual hours	6	10.0
Retired	4	6.7
Full-time education	1	1.7
Not working	1	1.7
*Education level*		
Educated to degree level or equivalent	29	48.3
Educated to A-levels or equivalent	18	30.0
Educated to GCSE-level (A*–C grades) or equivalent	13	21.7
*PrEP status*		
Starting PrEP for the first time (at recruitment)	11	18.3
Previously used PrEP	49	81.7
*Relationship status*		
Single	46	76.7
In a relationship	12	20.0
Married	2	3.3
*Sexual orientation*		
Gay man	56	93.3
Bisexual	3	5.0
Pansexual	1	1.7
*Sexual preferences*		
Has sex exclusively with men	59	98.3
Has sex with both men and women	1	1.7
Chronic health condition/s*		
At least one co-morbid health condition	27	45.0
Asthma/respiratory condition*	9	15.0
Mood disorder/mental health condition*	6	10.0
Digestive tract condition*	6	10.0
Other condition*	15	25.0
	Median	IQR
Age	35.5	28.0 to 46.0

*Participants may have more than one health condition. Mood disorder / mental health condition includes: Anxiety, depression, stress, and schizophrenia; Digestive tract condition includes: Gastro-oesophageal reflux disease (GORD), irritable bowel syndrome (IBS), stomach ulcer, coeliac disease, ulcerative colitis; Other condition includes: epilepsy, episodic ataxia, fibromyalgia, gout, psoriatic arthopathy, trigeminal neuralgia, high cholesterol, prostatitis, anaemia, epilepsy, hypertension, hydronephrosis, erectile dysfunction, hay fever, diabetes, acne, psoriasis

There were zero HIV seroconversions within our participants over the study period.

### Data availability

There were seven withdrawals through the study—four withdrew because they were no longer continuing to take PrEP, two because they moved out of Wales so could no longer access PrEP through the NHS in Wales, and one due to bereavement (Fig. 1). All seven participants contributed to the analysis up until the point of their withdrawal. Reported episodes of condomless vaginal sex were rare and limited to a small number of participants within the study. These data were therefore not analysed. Daily PrEP use and CAS data availability are outlined in Table SIV.

### Outcomes Measured Prior to the Introduction of Control Measures

#### PrEP Use

**Fig. 2:**
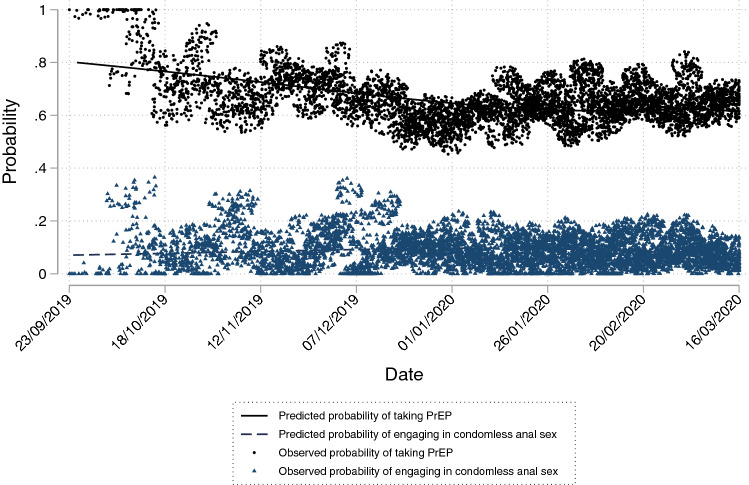
Overall predicted probabilities of taking PrEP and engaging in condomless anal sex during the pre-pandemic period*. * Solid black line indicates the estimated marginal probability of PrEP use from the PrEP use model. Navy dashed line indicates the estimated marginal probability of engaging in condomless anal sex from the CAS model. Grey circles indicate the observed marginal probabilities of PrEP use. Navy triangles indicate the observed marginal probabilities of condomless anal sex. Jitter effects have been applied to illustrate over-plotting

Prior to the introduction of control measures, PrEP was taken on 3791/5785 days (65.5%) and the probability of participants taking PrEP on a given day decreased steadily over time from around 0.8 to just over 0.6 (Table 2; Fig. 2). There was considerable variation in trajectories between participants (Fig. S1).

**Table 2:** Two-level logistic regression models of daily PrEP use (yes/no) over time

Variable	Odds ratio	Lower 95% CI	Upper 95% CI	z	p-value
Intercept	0.91	0.28	3.02	− 0.15	0.880
Time (1)	0.99	0.98	1.00	− 2.61	0.009
Time (2)	1.05	0.98	1.14	1.37	0.169
Time (3)	0.19	0.00	1138.20	− 0.37	0.709
Pandemic time	0.00	0.00	0.50	− 2.07	0.038
Time (1) × pandemic time	0.68	0.48	0.97	− 2.16	0.031
Time (2) × pandemic time	1.86	1.00	3.45	1.96	0.050
Time (3) × pandemic time	1.21	0.00	7911.05	0.04	0.966
*Covariances*					
Intercept	4.47	2.74	7.28		
Pandemic time	3.75	2.33	6.04		
Intercept × pandemic time	− 1.55	− 2.90	− 0.20		

Model based on 13,322 observations within 53 participants. Time was modelled as a cubic spline term (with knots at T = − 126, − 24, 64, and 184). An unstructured covariance matrix was specified and robust standard errors were estimated

**Fig. 3:**
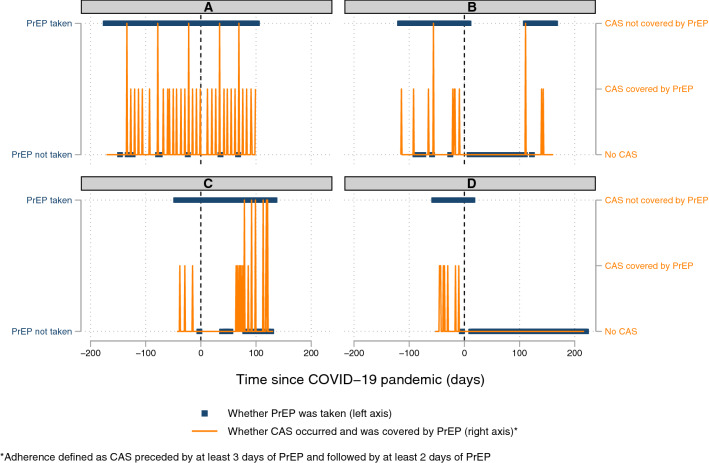
Panel plot of daily PrEP adherence and daily PrEP use over time for four participants (A to D)*. *Left-hand y-axis displays daily PrEP use (yes/no, blue hollow circles). Right-hand y-axis displays whether a CAS episode occurred and whether it was covered by daily PrEP according to our definition (orange lines). X-axis displays time in relation to the introduction of COVID-19 pandemic-related control measures

#### Sexual Behaviour

Participants reported engaging in CAS on 506/5559 days prior to the introduction of control measures (9.1% of observed days), with a mean number of CAS episodes on a given day of 0.17 (SD = 0.661) and a mean number of partners with whom a participant engaged in CAS on a given day was 0.14 (SD = 0.530), equating to approximately one CAS episode every 6 days and one partner per week, on average. There was a steady increase in the probability of a participant engaging in CAS prior to the introduction of control measures (from 0.07 at the beginning of the observation period to around 0.1 around 60 days prior to the introduction of control measures), before decreasing again to 0.07 as the introduction of control measures neared (Table 3; Fig. 2). Similar to PrEP use, there was considerable variation in CAS between participants (Figs. S1, S2).

**Table 3:** Two-level logistic regression models of condomless anal sex (yes/no) over time

Variable	Odds ratio	Lower 95% CI	Upper 95% CI	z	p-value
Intercept	0.11	0.06	0.20	− 7.00	< 0.001
Time (1)	1.00	1.00	1.01	1.46	0.141
Time (2)	0.96	0.92	0.99	− 2.28	0.022
Time (3)	1.24	1.06	1.44	2.67	0.007
Time (4)	0.74	0.60	0.91	− 2.90	0.004
Pandemic time	0.35	0.17	0.69	− 2.99	0.003
*Covariances*					
Intercept	1.02	0.59	1.76		
Pandemic time	1.03	0.50	2.15		
Intercept x Pandemic time	0.06	− 0.40	0.52		

Model based on 12,913 observations within 59 participants. Time was modelled as a cubic spline term (with knots at T = -122, -39, 23, 91, and 188). An unstructured covariance matrix was specified and robust standard errors were estimated

Models focusing on the number of times participants engaged in CAS and number of different partners displayed similar patterns to the dichotomous model (Tables SV, SVI; Figs. S3, S4).

#### PrEP Use and Sexual Behaviour

The model examining the relationship between prior PrEP use and subsequent CAS included 11,500 observations within 58 participants. We found that prior PrEP use was associated with almost two-fold higher odds of subsequent CAS (OR 1.93, 95% CI 1.16–3.23, z = 2.53, p = 0.011).

#### PrEP Adherence

PrEP adherence was assessed for 406 CAS episodes over 4801 days prior to the introduction of control measures. In daily PrEP users, PrEP use was adequate to cover 207 (51.0%) CAS episodes, with 10/49 (20.4%) participants taking daily PrEP to cover all CAS episodes. In our sensitivity analysis excluding participant data where there was suspected inconsistent use of the MEMS cap, PrEP adherence was assessed for 329 CAS episodes over 4,097 days and PrEP use was adequate to cover 205 (62.3%) CAS episodes.

### Impact of the COVID-19 Pandemic

The odds of participants taking PrEP on a given day were 56% lower following the introduction of control measures (OR 0.44, 95% CI 0.20 to 0.95, z = − 2.09, p = 0.037) (Table 2). As illustrated by Figure S5, there was a statistically significant difference in slopes pre- and during the pandemic [χ^2^(3) = 8.24, p-value for joint test of interaction terms = 0.041].

Similarly, the introduction of control measures was associated with a marked reduction in CAS (OR averaged over time during the pandemic = 0.35, 95% CI 0.17–0.69, z  =  − 2.99, p  = 0.003) (Table 3; Fig. S5). There was no evidence to suggest a difference in slopes pre- and during the pandemic [χ^2^(3) = 5.67, p-value for joint test of interaction terms = 0.129].

We found some indication that the association between prior PrEP use and subsequent CAS was higher following the introduction of control measures compared to the time prior to their introduction. However, this association was not statistically significant (OR for prior PrEP use × pandemic time interaction = 1.56, 95% CI 0.98–2.47, z = 1.88, p = 0.060).

Following the introduction of control measures, PrEP adherence was assessed for 311 CAS episodes over 6,076 days. In daily PrEP users, PrEP use was adequate to cover 88 (28.3%) CAS episodes (RR = 0.55, 95% CI 0.34–0.89, z = − 2.42, p = 0.015), with 4/46 (8.7%) participants taking daily PrEP to cover all CAS episodes. Our sensitivity analysis encompassed 214 CAS episodes over 4407 days and PrEP use was adequate to cover 78 (36.4%) CAS episodes (RR = 0.58, 95% CI 0.36–0.94, z = − 2.21, p = 0.027).

PrEP adherence varied between- and within-individuals over time, with some participants displaying high levels of PrEP adherence over time and others less so. Figure 3 and Table S7 illustrate the different patterns of PrEP use and PrEP adherence and different impacts of pandemic-related control measures within our dataset. For example, Participant A demonstrated high levels of daily PrEP use and CAS, most of which were covered by adequate amounts of PrEP, with no discernible change in these patterns following the introduction of control measures. Participant B demonstrated similarly high levels of daily PrEP use and less frequent CAS episodes prior to the introduction of control measures. Following their introduction, the participant paused their PrEP use and restarted when CAS behaviour resumed. Note their first reported CAS episode was not covered by an adequate amount of PrEP according to our definition. Participant C demonstrated more frequent CAS episodes and lower adherence following the introduction of control measures. Participant D demonstrated high levels of daily PrEP use and CAS prior to the introduction of control measures, with a cessation of both PrEP use and CAS following their introduction.

## Discussion

### Summary of Main Findings

In our study of PrEP users in Wales, we found considerable variation between- and within-individuals in their PrEP use and sexual risk behaviours over time. While our study indicated that PrEP users tailor their PrEP use according to their risk exposure (i.e. that prior PrEP use is associated with subsequent CAS), we found that only 51 to 63% of CAS episodes were covered by an adequate supply of PrEP among daily PrEP users.

The introduction of measures to control the transmission of SARS-CoV-2 was associated with marked reductions in both PrEP use and CAS episodes. We found some evidence to suggest that PrEP users were more likely to tailor their PrEP use according to their risk exposure (though this was not statistically significant), yet despite this only 28 to 36% of CAS episodes were covered by an adequate intake of PrEP among daily PrEP users.

### Strengths and Limitations

We used intensive longitudinal methods, benefitting from frequent measurement of observations within individuals over time, rather than relying on a measure collected at a single point in time or infrequent repeated observations. Measures collected at a single point in time would not allow for an understanding of temporal trends and may be more prone to recall bias and measurement error. Infrequent repeated observations may have lacked sensitivity to detect within-person changes and the interrelationship between PrEP use and sexual behaviour. We achieved high levels of follow-up for both PrEP use and sexual behaviour. By measuring PrEP use using an electronic monitor, we utilised a non-intrusive method to capture PrEP use which was unlikely to be subject to measurement reactivity in the same way that self-report and tablet count measurement approaches may be [31, 32]. By capturing CAS behaviour using brief weekly electronic surveys, we limited response burden both in terms of time spent completing questions as well as the recall period. We sampled participants across four of the seven health boards offering PrEP through the NHS in Wales, and included a nationally representative sample of participants covering 5% of all individuals accessing PrEP through the NHS in Wales at the time.

The study was designed to include all PrEP users. However, only MSM were included and the majority of participants were white. While this is largely representative of the individuals accessing PrEP through NHS Wales, the findings may not generalise to other key populations.

The weekly sexual behaviour surveys were intentionally kept brief to reduce response burden, but doing so may have limited the information that they provide. For example, while the number of CAS episodes and number of different partners were recorded, no differentiation between partner types was made [33]. Furthermore, the use of the MEMS relied on the assumption that each cap opening corresponding to an individual removing and ingesting one tablet. The levels of PrEP adherence found in our study may in part be explained by these two aspects.

Finally, while a key strength of this study is that it was set-up in advance of the COVID-19 pandemic, allowing for longitudinal measures of both PrEP use and CAS prior to and following the introduction of control measures, the number of observations available prior to their introduction (and hence our ability to study natural behaviour as per the original aim of the study), was limited.

### Comparisons with Existing Literature

Our finding of a gradual decrease in PrEP use over time is consistent with findings from a demonstration project in New South Wales, which indicated a decline in daily PrEP adherence over time across a range of measures [34]. However, while the study measured adherence in various ways, its definition was based on PrEP use only and not within the context of risk exposure. Adherence to PrEP was lower in our study than previously reported [35, 36]. This may be explained by different definitions of adequate PrEP intake and risk exposure, but also by the different geographical settings, HIV incidences and PrEP use measures (electronic monitor versus self-report). Our findings align with a recent survey of young sexual minority men in the US found PrEP use and sexual activity decreased during the COVID-19 pandemic, with over a third reporting CAS with a casual partner three-months or later after the introduction of pandemic-related control measures [37].

### Implications

Our work highlights varying levels of PrEP use and CAS over time, with PrEP adherence estimated to be lower than that typically found in other PrEP cohorts. Furthermore, the introduction of pandemic-related control measures was associated with a substantial reductions in all three outcomes. The reduction in both CAS and PrEP adherence implies that while reduced in number, participants engaging in CAS following the introduction of pandemic-related control measures were less likely to be covered by PrEP and therefore may be associated with greater risk of HIV-transmission. These findings may have implications for the health and wellbeing, and in particular the sexual wellbeing of PrEP users [38]. Furthermore, lower levels of PrEP adherence coupled with disruption to (or alteration of) sexual health services may pose a risk in terms of new HIV diagnoses and thus contribute to the wider threats across the HIV prevention cascade [39]. Policy decision-making and public health messaging should factor in the role of sexual wellbeing on future pandemic restrictions or other restrictions where both access to sexual health services and social interaction may be affected.

Further work is needed to investigate the within-individual behavioural determinants of PrEP use, CAS, and PrEP coverage over time; gain a deeper understanding of the experiences of PrEP and the impact that the pandemic had on PrEP use and sexual risk behaviours; determine the extent to which PrEP non-adherence in this study reflects genuine risk exposure or undocumented changes in relationship dynamics; understand the longer-term impact on the health and wellbeing of PrEP users as we emerge from the pandemic.

Greater emphasis should be placed on supporting long-term PrEP adherence, with post-exposure prophylaxis (PEP) offered in circumstances whereby CAS episodes are not covered by an adequate intake of PrEP.

## Conclusion

Daily PrEP use, CAS, and PrEP adherence is highly variable between- and within- MSM PrEP users in Wales over time. A high proportion of CAS episodes were not covered by adequate PrEP use, and the introduction of pandemic-related control measures was associated with substantial reductions in PrEP use, CAS, but most concerning PrEP adherence. The latter of these findings warrants further investigation to determine the risk associated with CAS episodes not covered by PrEP.

## Supplementary Information

Below is the link to the electronic supplementary material.

Supplementary file1 (DOCX 5429 KB)

## Data Availability

Data are available upon request.
