# Supplementary material for: PrEP Use, Sexual Behaviour, and PrEP Adherence Among Men who have Sex with Men Living in Wales Prior to and During the COVID-19 Pandemic
Source: AIDS Behav. 2022 Feb 19;26(8):2746–57. doi: 10.1007/s10461-022-03618-4 (PMC8857895; doi:10.1007/s10461-022-03618-4)
Supplement: Supplementary file 1 — Supplementary file1 (DOCX 5429 KB) [file 10461_2022_3618_MOESM1_ESM.docx]

**PrEP use, sexual behaviour, and PrEP adherence among men who have sex with men living in Wales prior to and during the COVID-19 pandemic: Supplementary Material**

**Table SI:** Sex quiz questions

| **Question** | **Question** | **Response options** |
| --- | --- | --- |
| 1 | Have you had sex without a condom in the past week? | No |
|  |  | Yes |
| 2 (if Yes selected in 1) | What type of sex have you had without a condom in the past week? [Select all that apply] | Anal |
|  |  | Vaginal |
| 3 (if Vaginal selected in 2) | For the condomless vaginal sex, did you know the HIV-status of your partner/s? | No / Not all of them |
|  |  | Yes - all of them and all HIV-negative |
|  |  | Yes – any that are HIV-positive had an undetectable viral load |
|  |  | Yes – and at least one HIV-positive and had a detectable viral load |
| 4 (if 1 = Yes) | Which days in the past week did you have condomless anal/vaginal sex? [Select all that apply] | 1 day ago |
|  |  | 2 days ago |
|  |  | 3 days ago |
|  |  | 4 days ago |
|  |  | 5 days ago |
|  |  | 6 days ago |
|  |  | 7 days ago |
| 5 (for each day selected and for CAS or CVS if HIV status was unknown or partner had a detectable viral load) | X day/s ago, how many times did you have CAS? | Integer response |
|  | X day/s ago, with how many partners did you have CAS? | Integer response |
|  | X day/s ago, how many times did you have CVS? | Integer response |
|  | X day/s ago, with how many partners did you have CVS? | Integer response |

**Extensions to binary PrEP use and CAS models:**

The models were extended in the following ways: i.) Including a time by pandemic interaction, as it was considered possible that trajectories in PrEP taking behaviour and CAS may differ pre- and intra-pandemic; ii.) We extended the binary CAS model to include PrEP use as a predictor, with the individual-specific probability of PrEP use over time also included in order to decompose between- and within-individual effects. PrEP use was lagged by one day, giving model estimates which can be interpreted as the association between PrEP use one-day prior and subsequent CAS. Lagged PrEP use was also included as a random effect. We chose a lag of one day as PrEP was generally prescribed daily and ongoing use is important to maintain effectiveness, with missed doses discouraged by clinic staff. We further explored whether the association was moderated by whether the observation occurred during pre- and intra-pandemic time by fitting interaction terms to PrEP use and our indicator for pandemic time.

**Comparison between PrEP use measures (MEMS versus self-report)**

We calculated the correlation between PrEP use according to the MEMS with self-reported PrEP use at each of the three follow-up time points. Across all participants, the correlation was 0·41 during the study entry to follow-up 1 period (n=52), 0·60 during the follow-up 1 to follow-up 2 period (n=43), and 0·55 during the follow-up 2 to follow-up 3 period (n=43). We considered participants may have used their MEMS cap inconsistently if, in a given time period, there was at least a 50 percentage point difference in the percentage of days on which PrEP was used according to the MEMS and the percentage of days where participants reported taking PrEP over the 30 days prior to the follow-up assessment. Excluding time periods where this was the case, the correlation between MEMS and self-report PrEP use was 0·60 during the study entry to follow-up 1 period (n=41), 0·89 during the follow-up 1 to follow-up 2 period (n=34), and 0·74 in the follow-up 2 to follow-up 3 period (n=26).

**Table SII:** Secondary outcome measures (behavioural)

| **Domain** | **Variable** | | **n** | **%** |
| --- | --- | --- | --- | --- |
| PrEP disclosure | Have you ever told anyone that you take PrEP? | No | 4 | 6·8 |
|  |  | Yes | 55 | 93·2 |
| PrEP stigma (personalised stigma) | People I care about stopped speaking to me after learning that I take PrEP | Strongly disagree | 38 | 69·1 |
|  |  | Disagree | 15 | 27·3 |
|  |  | Agree | 1 | 1·8 |
|  |  | Strongly agree | 1 | 1·8 |
|  | I have lost friends by telling them I take PrEP | Strongly disagree | 40 | 72·7 |
|  |  | Disagree | 13 | 23·6 |
|  |  | Agree | 1 | 1·8 |
|  |  | Strongly agree | 1 | 1·8 |
|  | I have been hurt by how people reacted to learning I take PrEP | Strongly disagree | 33 | 60·0 |
|  |  | Disagree | 14 | 25·5 |
|  |  | Agree | 7 | 12·7 |
|  |  | Strongly agree | 1 | 1·8 |
|  | I regret having told some people that I take PrEP | Strongly disagree | 35 | 63·6 |
|  |  | Disagree | 15 | 27·3 |
|  |  | Agree | 5 | 9·1 |
|  |  | Strongly agree | 0 | 0·0 |
|  | I have stopped socialising with some people due to their reactions when learning that I take PrEP | Strongly disagree | 40 | 72·7 |
|  |  | Disagree | 15 | 27·3 |
|  |  | Agree | 0 | 0·0 |
|  |  | Strongly agree | 0 | 0·0 |
| PrEP stigma (disclosure concerns) | Telling someone I take PrEP is risky | Strongly disagree | 23 | 38·3 |
|  |  | Disagree | 24 | 40·0 |
|  |  | Agree | 11 | 18·3 |
|  |  | Strongly agree | 2 | 3·3 |
|  | I work hard to keep my PrEP use a secret | Strongly disagree | 28 | 46·7 |
|  |  | Disagree | 27 | 45·0 |
|  |  | Agree | 4 | 6·7 |
|  |  | Strongly agree | 1 | 1·7 |
|  | I am very careful whom I tell that I take PrEP | Strongly disagree | 19 | 31·7 |
|  |  | Disagree | 21 | 35·0 |
|  |  | Agree | 16 | 26·7 |
|  |  | Strongly agree | 4 | 6·7 |
|  | In many areas of my life, no one knows I take PrEP | Strongly disagree | 12 | 20·0 |
|  |  | Disagree | 19 | 31·7 |
|  |  | Agree | 21 | 35·0 |
|  |  | Strongly agree | 8 | 13·3 |
|  | I worry about people discriminating against me because I take PrEP | Strongly disagree | 19 | 31·7 |
|  |  | Disagree | 23 | 38·3 |
|  |  | Agree | 15 | 25·0 |
|  |  | Strongly agree | 3 | 5·0 |
|  | I worry that people will assume that because I take PrEP I have sex with lots of people | Strongly disagree | 9 | 15·0 |
|  |  | Disagree | 17 | 28·3 |
|  |  | Agree | 27 | 45·0 |
|  |  | Strongly agree | 7 | 11·7 |
|  | I worry that people will assume that because I take PrEP I am HIV positive | Strongly disagree | 21 | 35·0 |
|  |  | Disagree | 23 | 38·3 |
|  |  | Agree | 14 | 23·3 |
|  |  | Strongly agree | 2 | 3·3 |
| HIV risk perceptions | Thinking about the type of sex you have had in the past month, if this were to continue in a similar way for the next year, what do you think your chances would be of becoming infected with HIV **if you were not taking PrEP?** | No chance: no possibility of becoming infected with HIV | 5 | 8·3 |
|  |  | A small chance: could happen but not likely | 26 | 43·3 |
|  |  | A moderate chance: some possibility of becoming infected with HIV | 22 | 36·7 |
|  |  | A high chance: likely to become infected with HIV | 7 | 11·7 |
|  | Thinking about the type of sex you have had in the past month, if this were to continue in a similar way for the next year, what do you think your chances would be of becoming infected with HIV **if you were taking PrEP?** | No chance: no possibility of becoming infected with HIV | 21 | 35·0 |
|  |  | A small chance: could happen but not likely | 34 | 56·7 |
|  |  | A moderate chance: some possibility of becoming infected with HIV | 4 | 6·7 |
|  |  | A high chance: likely to become infected with HIV | 1 | 1·7 |
| Attitudes (instrumental and experiential respectively) | Taking PrEP as prescribed would be | Bad | 0 | 0·0 |
|  |  | 2 | 1 | 1·7 |
|  |  | 3 | 2 | 3·3 |
|  |  | 4 | 2 | 3·3 |
|  |  | Good | 55 | 91·7 |
|  | Taking PrEP as prescribed would be | Pleasant | 30 | 50·0 |
|  |  | 2 | 15 | 25·0 |
|  |  | 3 | 14 | 23·3 |
|  |  | 4 | 0 | 0·0 |
|  |  | Unpleasant | 1 | 1·7 |
| Perceived norms (injunctive and descriptive norms respectively) | Most people who are important to me approve of me taking PrEP as prescribed | Strongly agree | 31 | 52·5 |
|  |  | 2 | 14 | 23·7 |
|  |  | 3 | 9 | 15·3 |
|  |  | 4 | 0 | 0·0 |
|  |  | Strongly disagree | 0 | 0·0 |
|  |  | N/A - nobody knows that I take PrEP | 5 | 8·5 |
|  | Most people who are like me take PrEP as prescribed | Unlikely | 2 | 3·4 |
|  |  | 2 | 4 | 6·8 |
|  |  | 3 | 17 | 28·8 |
|  |  | 4 | 17 | 28·8 |
|  |  | Likely | 19 | 32·2 |
| Perceived behavioural control (capacity and autonomy respectively) | I am confident that I can take PrEP as prescribed | True | 53 | 88·3 |
|  |  | 2 | 4 | 6·7 |
|  |  | 3 | 2 | 3·3 |
|  |  | 4 | 0 | 0·0 |
|  |  | False | 1 | 1·7 |
|  | Taking PrEP as prescribed is up to me | Strongly disagree | 6 | 10·0 |
|  |  | 2 | 3 | 5·0 |
|  |  | 3 | 5 | 8·3 |
|  |  | 4 | 5 | 8·3 |
|  |  | Strongly agree | 41 | 68·3 |
| Intentions | I intend to continue taking PrEP as prescribed | Likely | 56 | 93·3 |
|  |  | 2 | 2 | 3·3 |
|  |  | 3 | 1 | 1·7 |
|  |  | 4 | 1 | 1·7 |
|  |  | Unlikely | 0 | 0·0 |
| Self-regulation (monitoring intake, monitoring supply, responding to missed doses respectively) | I watch carefully that I take PrEP as prescribed | Never | 0 | 0·0 |
|  |  | 2 | 0 | 0·0 |
|  |  | 3 | 3 | 5·1 |
|  |  | 4 | 11 | 18·6 |
|  |  | Always | 45 | 76·3 |
|  | I make sure that I get my new PrEP prescription at the clinic before my last prescription is finished | Never | 0 | 0·0 |
|  |  | 2 | 1 | 1·7 |
|  |  | 3 | 2 | 3·4 |
|  |  | 4 | 6 | 10·3 |
|  |  | Always | 49 | 84·5 |
|  | If I notice that I have not taken PrEP, I think about what the reason for that was and how I can prevent that from happening again | Never | 5 | 8·6 |
|  |  | 2 | 2 | 3·4 |
|  |  | 3 | 3 | 5·2 |
|  |  | 4 | 12 | 20·7 |
|  |  | Always | 36 | 62·1 |
| Action planning (frequency, timing, and location respectively) | Looking at the next four-weeks, I know exactly how often I will take PrEP | Strongly disagree | 1 | 1·7 |
|  |  | 2 | 1 | 1·7 |
|  |  | 3 | 2 | 3·4 |
|  |  | 4 | 4 | 6·8 |
|  |  | Strongly agree | 51 | 86·4 |
|  | Looking at the next four-weeks, I know exactly at what time I will take PrEP | Strongly disagree | 1 | 1·7 |
|  |  | 2 | 4 | 6·7 |
|  |  | 3 | 6 | 10·0 |
|  |  | 4 | 14 | 23·3 |
|  |  | Strongly agree | 35 | 58·3 |
|  | Looking at the next four-weeks, I know exactly where I will take PrEP | Strongly disagree | 0 | 0·0 |
|  |  | 2 | 3 | 5·0 |
|  |  | 3 | 8 | 13·3 |
|  |  | 4 | 7 | 11·7 |
|  |  | Strongly agree | 42 | 70·0 |
| Anticipated regret (anticipated regret and anticipated affective reaction respectively) | If I were to miss a dose of PrEP I would feel | Absolutely no regret | 1 | 1·7 |
|  |  | 2 | 5 | 8·3 |
|  |  | 3 | 22 | 36·7 |
|  |  | 4 | 16 | 26·7 |
|  |  | Very much regret | 16 | 26·7 |
|  | If I were to miss a dose of PrEP I would feel | Absolutely not upset | 6 | 10·0 |
|  |  | 2 | 8 | 13·3 |
|  |  | 3 | 23 | 38·3 |
|  |  | 4 | 11 | 18·3 |
|  |  | Very much upset | 12 | 20·0 |

**Table SIII:** STI diagnoses and healthcare resource use in the 3 months prior to study entry

| **Variable** | | **n** | | **%** | |
| --- | --- | --- | --- | --- | --- |
| STI diagnoses in past three months | No | 48 | | 80·0 | |
|  | Yes | 12 | | 20·0 | |
| Type of STI* | Oral chlamydia trachomatis | 1 | | 1·7 | |
|  | Rectal chlamydia | 3 | | 5·0 | |
|  | Chlamydia (site not specified) | 2 | | 3·3 | |
|  | Oral gonorrhoea | 1 | | 1·7 | |
|  | Gonorrhoea (site not specified) | 1 | | 1·7 | |
|  | Non-specific  genital infection | 1 | | 1·7 | |
|  | Giardia | 1 | | 1·7 | |
|  | Syphilis | 3 | | 5·0 | |
| **Variable** | | **Mean** | **SD** | **Min** | **Max** |
| Healthcare contacts in past 3 months | This sexual health clinic | 2 | 1 | 1 | 7 |
|  | Other sexual health clinic | 0 | 0 | 0 | 2 |
|  | GP at surgery | 1 | 1 | 0 | 5 |
|  | Nurse at surgery | 0 | 1 | 0 | 3 |
|  | GP at home | 0 | 1 | 0 | 10 |
|  | Nurse at home | 0 | 1 | 0 | 10 |
|  | GP on telephone | 0 | 1 | 0 | 5 |
|  | Nurse on telephone | 0 | 0 | 0 | 0 |
|  | Doctor at hospital | 0 | 2 | 0 | 10 |
|  | Nurse at hospital | 0 | 1 | 0 | 4 |
|  | Telephone consultation  with the NHS | 0 | 0 | 0 | 0 |
|  | NHS 111 | 0 | 0 | 0 | 2 |
|  | A&E | 0 | 0 | 0 | 2 |
|  | Hospital clinic | 0 | 1 | 0 | 5 |

*Some participants had more than one STI diagnosed.

**Table SIV:** PrEP use and CAS data availability

|  | **Prior to the introduction of control measures** | | **Following the introduction of control measures** | |
| --- | --- | --- | --- | --- |
|  | **Frequency** | **%** | **Frequency** | **%** |
| **Total number of participants** | **60** | **100·0** | **57** | **100·0** |
| **Total number of potentially observed days** | **6,565** | **100·0** | **9,442** | **100·0** |
| Daily PrEP data (participants) | 53 | 88·3 | 49 | 86·0 |
| Daily PrEP data (days) | 5,785 | 88·1 | 7,537 | 79·8 |
| Daily CAS data (participants) | 58 | 96·7 | 55 | 96·5 |
| Daily CAS data (days) | 5,559 | 84·7 | 7,354 | 77·9 |
| PrEP adherence (participants)* | 49 | 81·7 | 46 | 80·7 |
| PrEP adherence (days)* | 4,801 | 73·1 | 6,076 | 64·4 |

*PrEP adherence analysed for participants who were following a daily PrEP regimen only.

**Figure S1:** Predicted probability of taking PrEP and engaging in condomless anal sex over the study period by participant*

*Panel plots created for participants with data pre- and during pandemic times. Solid black lines indicate the estimated probability of taking PrEP from the PrEP use model for each individual. Navy lines indicate the estimated probability of engaging on condomless anal sex from the CAS model for each individual. Time is defined as time since the onset of pandemic-related control measures (time zero = 16/03/2021). The red vertical dashed line indicates the date of the introduction of pandemic-related control measures across Wales.

**Figure S2:** Predicted probability of engaging in condomless anal sex over the study period by participant

*Panel plots created for participants with data pre- and during pandemic times. Thick black lines indicate the probability estimated from the model for each individual. Time is defined as time since the onset of pandemic-related control measures (time zero = 16/03/2021). The red vertical dashed line indicates the date of the introduction of pandemic-related control measures across Wales.

**Table SV:** Two-level ordered logistic regression models of the number of times participants engaged in condomless anal sex (0/1/2/3 or more) over time*

| **Variable** | **Odds ratio** | **Lower 95% CI** | **Upper 95% CI** | **z** | **p-value** |
| --- | --- | --- | --- | --- | --- |
| Time (1) | 1·00 | 1·00 | 1·01 | 1·46 | 0·145 |
| Time (2) | 0·96 | 0·92 | 0·99 | -2·26 | 0·024 |
| Time (3) | 1·22 | 1·05 | 1·41 | 2·63 | 0·009 |
| Time (4) | 0·75 | 0·62 | 0·92 | -2·84 | 0·005 |
| Pandemic time | 0·32 | 0·16 | 0·64 | -3·23 | 0·001 |
|  |  |  |  |  |  |
| **Cut points for ordinal model** | **Coefficient** | **Lower 95% CI** | **Upper 95% CI** |  |  |
| Cut point 1 | 2·26 | 1·64 | 2·88 |  |  |
| Cut point 2 | 3·37 | 2·62 | 4·12 |  |  |
| Cut point 3 | 4·28 | 3·51 | 5·06 |  |  |
|  |  |  |  |  |  |
| **Covariances** | **Coefficient** | **Lower 95% CI** | **Upper 95% CI** |  |  |
| Intercept | 1·07 | 0·65 | 1·75 |  |  |
| Pandemic time | 1·02 | 0·49 | 2·10 |  |  |

*Model based on 12,913 observations within 59 participants. Time was modelled as a cubic spline term (with knots at T = -122, -39, 23, 91, and 188). An unstructured covariance matrix was specified and robust standard errors were estimated.

**Table SVI:** Two-level ordered logistic regression models of the number sexual partners engaged in condomless anal sex (0/1/2 or more) over time*

| **Variable** | **Odds ratio** | **Lower 95% CI** | **Upper 95% CI** | **z** | **p-value** |
| --- | --- | --- | --- | --- | --- |
| Time (1) | 1·00 | 1·00 | 1·01 | 1·52 | 0·129 |
| Time (2) | 0·95 | 0·92 | 0·99 | -2·43 | 0·015 |
| Time (3) | 1·24 | 1·07 | 1·45 | 2·83 | 0·005 |
| Time (4) | 0·73 | 0·60 | 0·90 | -3·04 | 0·002 |
| Pandemic time | 0·36 | 0·18 | 0·70 | -3·03 | 0·002 |
|  |  |  |  |  |  |
| **Cut points for ordinal model** |  |  |  |  |  |
| Cut point 1 | 2·24 | 1·61 | 2·86 |  |  |
| Cut point 2 | 3·85 | 3·14 | 4·56 |  |  |
|  |  |  |  |  |  |
| **Covariances** |  |  |  |  |  |
| Intercept | 1·05 | 0·65 | 1·69 |  |  |
| Pandemic time | 1·01 | 0·48 | 2·10 |  |  |

*Model based on 12,913 observations within 59 participants. Time was modelled as a cubic spline term (with knots at T = -122, -39, 23, 91, and 188). An unstructured covariance matrix was specified and robust standard errors were estimated.

**Figure S3:** Model predictions and observed mean number of times participants engaged in CAS on a given day

**Figure S4:** Model predictions and observed mean number of partners participants engaged in CAS on a given day

**Table SVII:** Summary statistics for four daily PrEP users

| **ID** | **Prior to the introduction of control measures** | | | | **Following the introduction of control measures** | | | |
| --- | --- | --- | --- | --- | --- | --- | --- | --- |
|  | **Number of days observed** | **% of days PrEP used** | **Number of CAS episodes** | **PrEP adherence %** | **Number of days observed** | **% of days PrEP used** | **Number of CAS episodes** | **PrEP adherence %** |
| A | 103 | 96·1 | 13 | 84·6 | 173 | 93·1 | 18 | 83·3 |
| B | 117 | 89·7 | 8 | 87·5 | 166 | 37·3 | 3 | 66·7 |
| C | 45 | 95·6 | 5 | 60·0 | 135 | 82·2 | 14 | 57·1 |
| D | 55 | 96·4 | 8 | 100·0 | 222 | 6·3 | 0 | N/A |

**Figure S5:** Overall predicted probabilities of taking PrEP and engaging in condomless anal sex over the study period*

* Solid black line indicates the estimated marginal probability of PrEP use from the PrEP use model. Navy dashed line indicates the estimated marginal probability of engaging in condomless anal sex from the CAS model. Grey circles indicate the observed marginal probabilities of PrEP use. Navy triangles indicate the observed marginal probabilities of condomless anal sex. Jitter effects have been applied to illustrate over-plotting. Time was defined as time since the onset of pandemic-related control measures (time zero = 16/03/2021). The red vertical dashed line indicates the date of the introduction of pandemic-related control measures across Wales.
